# Theoretical investigations of two-dimensional intrinsic magnets derived from transition-metal borides M_3_B_4_ (M = Cr, Mn, and Fe)

**DOI:** 10.1080/14686996.2024.2404384

**Published:** 2024-10-09

**Authors:** Chunmei Ma, Shiyao Wang, Chenguang Gao, Junjie Wang

**Affiliations:** a State Key Laboratory of Solidification Processing, Northwestern Polytechnical University, Xi’an, China; b School of Materials Science and Engineering, Northwestern Polytechnical University, Xi’an, China

**Keywords:** Two-dimensional transition metal borides, magnetic coupling, mechanical property, First-principles calculations

## Abstract

Two-dimensional (2D) magnetic materials with high critical temperatures (*T*_*C*_) and robust magnetic anisotropy energies (MAE) hold significant potential for spintronic applications. However, most of 2D magnetic materials are derived from the van der Waals (vdW) layered bulks, which greatly limits the synthesis of 2D magnetic materials. Here, 2D M_3_B_4_ (M = Cr, Mn, and Fe; B = Boron), derived from hexagonal and orthorhombic M_3_AlB_4_ phases by selectively etching Al layers, was studied for its structural stability, electronic structure, and magnetic properties. By utilizing *ab initio* calculations and Monte Carlo simulations, we found that the orthorhombic Cr_3_B_4_ shows ferromagnetic (FM) metal and possesses an in-plane magnetic easy axis, while the remaining hexagonal and orthorhombic M_3_B_4_ structures exhibit antiferromagnetic (AFM) metals with a magnetic easy axis which is perpendicular to the two-dimensional plane. The critical temperatures of these 2D M_3_B_4_ structures are found to be above the 130 K. Notably, the ort-Mn_3_B_4_ possesses highest *T*_*C*_ (~600 K) and strongest MAE (~220 µeV/atom) among these borides-based 2D magnetic materials. Our findings reveal that the 2D M_3_B_4_ compounds exhibit much better resistance to deformation compared to M_2_B_2_ MBenes and other 2D magnetic materials. The combination of high critical temperature, robust MAE, and excellent mechanical properties makes 2D Mn_3_B_4_ monolayer exhibits a favorable potential for spintronic applications. Our research also sheds light on the magnetic coupling mechanism of 2D M_3_B_4_, providing valuable insights into its fundamental characteristics.

## IMPACT STATEMENT

Two-dimensional magnetic hexagonal and orthorhombic M_3_B_4_ compounds with significant stiffness to preserve their magnetic properties were discovered using DFT calculations and MC simulations.

## GRAPHICAL ABSTRACT

**Figure:**
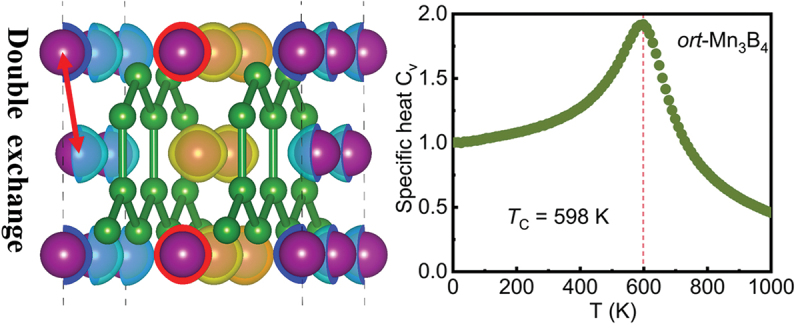

## 1. Introduction

In recent years, there has been a notable increase in the attention given to the study of magnetic materials with two-dimensional (2D) properties, primarily due to their potential applications in data storage and spintronic devices [1–3]. However, the Mermin-Wagner theory states that the isotropic 2D Heisenberg model lacks spontaneous polarization at finite temperatures due to thermal fluctuations [4]. Hence, to establish magnetic order within 2D material systems, it is crucial to possess a substantial magnetic anisotropy energy (MAE) that can effectively resist the detrimental impacts induced by thermal fluctuations.

In 2017, there were novel 2D intrinsic ferromagnetic materials such as Cr_2_Ge_2_Te_6_ (*T*_*C*_ = ∼30 K) [5,6] and CrI_3_ (*T*_*C*_ = ∼45 K) [7,8] opened new avenues for the study of single-layer and few-layer 2D magnetic materials. However, these materials possess very low critical temperatures and exhibit poor air stability, limiting their practical applications. In 2018, the synthesis of the 2D intrinsic magnet Fe_3_GeTe_2_ (*T*_*C*_ = ∼205 K) [9] advanced the study of 2D magnetic materials and provided a novel platform for spintronics development. In particular, there has been a significant amount of theoretical and experimental efforts dedicated to discovering 2D intrinsic ferromagnetic materials with substantial MAE, high critical temperature, and large magnetic moment (M), *e.g*., CrTe_2_ (*T*_*C*_ = ∼300 K; *M*  = ~3 µB/atom) [10], CrX_2_ (X = O, S, and Se) [11], and VO_2_ [12].

MXenes, characterized by the general formula M_n + 1_X_n_T_x_ (*M* = transition metal, C = carbon and/or nitrogen, *T* = F, Cl, OH or O, *n* = 1–3), which is called 2D transition metal carbides and carbonitrides synthesized through chemical etching of A layers from bulk MAX phases [13–15]. Notably, density functional theory (DFT) calculations have demonstrated that several bare and surface-terminated MXenes exhibit excellent magnetic properties, such as Ti_3_C_2_ [16], Cr_2_C [17] and Cr_2_NT_2_ (*T* = O, OH, or F) [18]. In 2015, a new class of ternary transition metal borides with an orthorhombic symmetric structure, known as MAB phase (space group *Cmmm*), was discovered [19]. These borides, denoted as (MB)_2_Al_y_(MB_2_)_x_, have similar structural with the well-known MAX phases. Building upon this discovery, MBenes were introduced as boride analogs of MXenes by selectively etching A layers from the MAB phases [20–22]. In 2019, Wang et al. predicted the existence of a hexagonal MAB phase of Ti_2_InB_2_ (space group P_6m2) and experimentally obtained TiB by exfoliating the indium layer [23]. More recently, Miao et al. have theoretically predicted and then experimentally synthesized several hexagonal MAB phases, including Hf_2_InB_2_, V_3_PB_4_, and Hf_2_PbB [24,25]. Notably, HfBO, a new hexagonal MBene, was achieved through chemical etching the In atoms of Hf_2_InB [24]. To distinguish between MAB phases with different symmetries, the hexagonal and orthorhombic MAB phases are referred to as *h*-MAB and *ort*-MAB, respectively. The resulting products after stripping the A-layer of *h*-MAB (or *ort*-MAB) phases are called *h*-MBenes (or *ort*-MBenes).

MBenes have emerged as highly promising candidates for low-dimensional magnetic materials due to their interaction with the unpaired *d* electrons in the transition metal element [20]. Although extensive studies have been conducted on the magnetic properties of 2D M_2_B_2_ derived from M_2_AB_2_ type phases [26–28], the electronic and magnetic properties of another important class of 2D MBenes, M_3_B_4_ compounds, derived from M_3_AB_4_ type phases have yet to be explored.

In this study, we conducted spin-polarized density functional theory calculations to investigate the structural, magnetic, and electronic properties of *h*-M_3_B_4_ and *ort*-M_3_B_4_ (*M* = Cr, Mn, Fe). Our findings revealed that M_3_B_4_ MBenes possess excellent mechanical properties to maintain a free-standing planar structure and magnetic states in external environments. Moreover, it was found that five intrinsic 2D magnets, namely *h*-Cr_3_B_4_, *h*-Mn_3_B_4_, *h*-Fe_3_B_4_, *ort*-Cr_3_B_4_, and *ort*-Mn_3_B_4_, exhibit remarkable structural stability. Among these compounds, *ort*-Cr_3_B_4_ demonstrates ferromagnetic behaviour with an in-plane magnetic easy axis. In contrast, *h*-Cr_3_B_4_, *h*-Mn_3_B_4_, *h*-Fe_3_B_4_, and *ort*-Mn_3_B_4_ are identified as Ising-type antiferromagnets with out-of-plane magnetic easy axis. This distinction in magnetic properties showcases the diverse behaviour observed in these materials. Furthermore, employing Monte Carlo simulations, the critical temperatures of 2D M_3_B_4_ are predicted to be above the 130 K, surpassing the critical temperature of CrI_3_ (45 K). Notably, the *ort*-Mn_3_B_4_ possesses highest *T*_*C*_ (~600 K) and strongest MAE (~220 µeV/atom) among these borides-based 2D magnetic materials, which make it exhibit a favorable potential for spintronic applications.

## 2. Computational methods

The spin-polarized density functional theory (DFT) method was utilized for all calculations in this study, which were carried out through the implementation of the Vienna ab-initio simulation package (VASP) [29]. The interaction between electrons and nuclei was described using the projector augmented wave (PAW) method [30]. The electronic exchange and correlation interactions are treated with the generalized gradient approximation (GGA) presented by the Perdew–Burke–Ernzerhof (PBE) formula [31]. Due to the presence of strong correlation effects in transition-metal atoms, GGA is insufficient to accurately describe the electronic and magnetic properties. Therefore, Dudarev’s PBE+*U* approach was utilized to incorporate on-site Coulomb interactions (*U*_eff_ = *U-J*) in order to obtain suitable magnetic solutions in metallic transition metal systems [32–34]. Changing the *U*_eff_ value from 2.0 eV to 4.0 eV yields the same magnetic ground state for all materials (Table S3) to validate the rationality of *U*_eff_. Previous studies have demonstrated that a small *U*_eff_ value can provide the appropriate magnetic solution for metallic transitions [35,36]. Therefore, *U*_eff_ = 2.0 eV was adopted in subsequent calculations. The plane-wave cutoff energy was set to 520 eV, and a 20 Å vacuum region was included to simulate 2D systems. During the geometric optimization process, structures were relaxed until their maximum force on atoms was less than 0.01 eV/Å, and the energy convergence criterion was set to 10^−6^ eV. A Г-centered *k*-point sampling of 15 × 15 × 1 was employed [37].

In order to ensure the dynamic stability of the studied 2D structures, phonon dispersion calculations were performed using a 2 × 2 × 1 supercell with the Phonopy code [38] within the framework of density functional perturbation theory (DFPT) [39]. Furthermore, Born-Oppenheimer *ab* initio molecular dynamics (AIMD) simulation was conducted in a 3 × 3 × 1 supercell at 600 K, employing a time step of 2.2 fs for a duration of 11 ps. Simulations were performed using the Nosé algorithm to control the temperature [40]. For the investigation of different magnetic configurations, a 2 × 2 × 1 supercell was utilized and constructed 4 (5) antiferromagnetic orders for *h*-M_3_B_4_ (*ort*-M_3_B_4_) using the Supercell package [41]. A classic Monte-Carlo (MC) simulation was conducted to determine the critical temperatures of M_3_B_4_ structures in this study, employing the ESpinS package [42]. A supercell with dimensions of 40 × 40 × 1 was utilized, allowing for random rotations in all possible directions for each spin. To ensure accuracy, the simulations for the 2D systems ran for 1 × 10^6^ steps to reach thermal equilibrium. By detecting the maximum point on the thermodynamic specific heat C_V_ graph, one can estimate the critical temperature at which a system achieves equilibrium under a certain temperature [43]. Furthermore, we employed a larger supercell of 60 × 60 × 1 and obtained the same transition temperatures for the magnetic phases to validate our MC simulations.

## 3. Results and discussion

### 3.1. Structural stability of 2D M_3_B_4_ compounds

**Figure 1.**
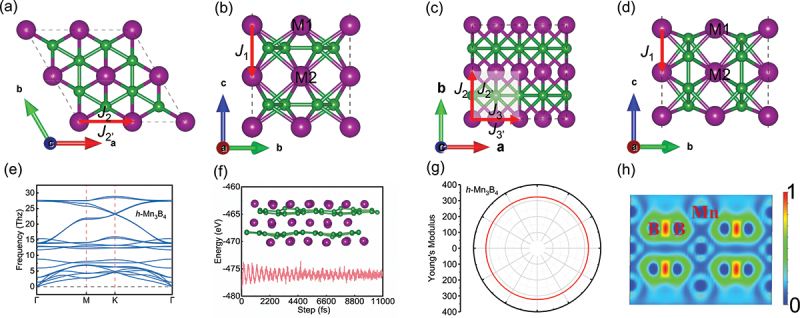
Structures and stabilities of 2D *h*-M_3_B_4_ and *ort*-M_3_B_4_. (a-d) top (a and c) and side views (b and d) of *h*-M_3_B_4_ (a and b) and *ort*-M_3_B_4_ (c and d), M represents Cr, Mn, and Fe; (e) phonon spectra of *h*-Mn_3_B_4_ monolayer; (f) calculated energy profile and the relaxed structure of the *h*-Mn_3_B_4_ monolayer obtained by an AIMD simulation of 11 ps at the temperature of 600 K; (g) calculated Young’s modulus of *h*-Mn_3_B_4_; (h) the cross-section of calculation electron localization function for *h*-Mn_3_B_4_ monolayer along the (110) plane. Spin exchange paths are shown in (a–d) with red solid arrow. The shadowed area in (a-d) indicates the unit cell. M1 and M2 represent transition metal atoms in the surface and middle layers, respectively. B is boron atom.

First, the crystal structures of *h*-M_3_B_4_ and *ort*-M_3_B_4_ were investigated, where M represents the transition metals Cr, Mn, and Fe. Figures 1(a-d) illustrate that these 2D structures consist of three layers of transition metal atoms. Moreover, *h*-M_3_B_4_ (with space group *P6/mmm*) exhibits a 2D graphene-like boron sheet, while *ort*-M_3_B_4_ (with space group *Pmmm*) features double triple chains of boron atoms. The surface atoms (M1) are surrounded by six neighboring boron atoms, whereas the interlayer atoms (M2) have 12 neighboring boron atoms. Table S1 provides detailed information on the structural parameters of *h*-M_3_B_4_ and *ort*-M_3_B_4_ 2D structures with different transition metals (*M* = Cr, Mn, and Fe).

To assess the structural stabilities of *h*-M_3_B_4_ and *ort*-M_3_B_4_ 2D structures, the phonon dispersion, molecular dynamics, and elastic constants of the material were calculated. Figure 1e and Figure S1 show phonon dispersions of *h*-M_3_B_4_ and *ort*-M_3_B_4_ (*M* = Cr, Mn, and Fe) at 0 K, which were calculated to determine their dynamic stability. Neither 2D *h*-M_3_B_4_ (*M* = Cr, Mn, and Fe) nor *ort*-M_3_B_4_ (*M* = Cr, and Mn) showed imaginary vibrational frequencies throughout their Brillouin Zones, showing their dynamic stability. AIMD simulations were further performed to evaluate the thermal stabilities of these five 2D materials at 600 K. As shown in Figure 1f and Figure S2, the energy fluctuated around the equilibrium state, demonstrating that all 2D structures remained stable at 600 K. Notably, no geometric reconstruction or broken bonds were observed during the entire process, underscoring the excellent thermal stabilities of these structures. In addition, the mechanical stabilities of these five materials were verified by calculating the elastic constants, as presented in Table 1. It is evident that these materials satisfy the Born mechanical stability criteria, with values of (C_11_, C_22_, C_66_) > 0 and C_11_·C_22_ > C_12_^2^ [44]. This further confirms the mechanical stability of these five 2D materials.

**Table 1.** Elastic properties of *h*-M_3_B_4_ and *ort*-M_3_B_4_ (*M* = Cr, Mn, Fe), including elastic constants *C*_*ij*_ (in N m^−1^), Young’s modulus *Y*_*x*_, *Y*_*y*_ (in N m^−1^), Poisson’s ratio *ϑ*_*x*_, *ϑ*_*y*_ (dimensionless) along the x, y directions, and out-of-plane deformation *h/L*.

	*C*_*11*_	*C*_*12*_	*C*_*22*_	*C*_*66*_	*Y*_*x*_	*Y*_*y*_	*ϑ*_*x*_	*ϑ*_*y*_	*h/L*
*h*-Cr_3_B_4_	319.7	40.2	323.2	140.3	314.7	314.7	0.12	0.12	2.33 × 10^−4^
*ort*-Cr_3_B_4_	279.7	87.3	372.3	133.3	259.2	345.0	0.23	0.31	2.11 × 10^−4^
*h*-Mn_3_B_4_	359.5	115.2	359.5	122.1	311.5	311.5	0.32	0.32	2.38 × 10^−4^
*ort*-Mn_3_B_4_	238.5	89.2	292.0	103.3	211.2	258.6	0.31	0.37	2.52 × 10^−4^
*h*-Fe_3_B_4_	328.0	114.0	328.0	107.0	288.4	288.4	0.35	0.35	2.47 × 10^−4^
Graphene	352.7	60.9	352.7	145.9	342.2	342.2	0.17	0.17	2.33 × 10^−4^

The in-plane Young’s modulus (*Y*) and Poisson’s ratio (*υ*) were calculated after considering the angle between directions and the *a* axis (Figure S3(f)). These calculations were performed using the following equations:

(1) $$Y\left(\theta \right)=\;\frac{C_{11}C_{22}\;-C_{12}^{2}\;}{C_{11}\mathrm{si}n4\theta +\mathrm{Asi}n2\mathrm{\theta co}s2\theta +\;C_{22}\mathrm{co}s4\theta }$$

(2) $$\vartheta \left(\theta \right)=\;\frac{C_{12}\mathrm{si}n4\theta -\mathrm{Bsi}n2\mathrm{\theta co}s2\theta +\;C_{12}\mathrm{co}s4\theta }{C_{11}\mathrm{si}n4\theta +\mathrm{Asi}n2\mathrm{\theta co}s2\theta +\;C_{22}\mathrm{co}s4\theta }$$

where $A=\frac{C_{11}C_{22}-C_{12}^{2}}{C_{66}}-2C_{12}$ and $B=C_{11}+C_{22}-\frac{C_{11}C_{22}-C_{12}^{2}}{C_{66}}$. The Figures S3 and S4 illustrate the orientation-dependent *Y(θ)* and *ϑ(θ)* values, respectively. It has been found that *h*-M_3_B_4_ (*M* = Cr, Mn, and Fe) exhibits a high isotropy in Young’s modulus and Poisson’s ratio, while those of *ort*-M_3_B_4_ (*M* = Cr and Mn) display anisotropy. Specifically, in the Cartesian [010] direction, the Young’s modulus and Poisson’s ratio of *ort*-M_3_B_4_ (*M* = Cr and Mn) reach their maximum values (Figures S3(d-e) and Figures S4(d-e)). Furthermore, we calculated Young’s modulus along the *x*- and *y*-axes, namely, *Y*_*x*_ (*Y*_*y*_) and *ϑ*_*x*_ (*ϑ*_*y*_), respectively (Table 1). For these five monolayers, their Young’s modulus is large enough to be comparable with graphene [44]. This indicates that M_3_B_4_ materials possess sufficient stiffness, which can be attributed to their strong bond interactions. Compared to common magnetic 2D materials like CrX_3_ (*Y*_2D_ ≈30 N/m) [45] and CrXTe_3_ (*Y*_2D_ ≈40 N/m) [46], the significant stiffness of M_3_B_4_ makes it resistant to deformation and better able to preserve its magnetic properties in external environments. At the same time, the mechanical parameters of *h*-M_2_B_2_ and *ort*-M_2_B_2_ (*M* = Cr, Mn, Fe) were also calculated for comparison (Table S2), which closely aligns with previous calculations [22]. Our calculations demonstrate that the elastic constant and Young’s modulus of M_3_B_4_ are even larger than those of the 2D M_2_B_2_ (*Y*_2D_ ≈200 N/m).

In addition, the elasticity theory suggests that the out-of-plane deformation can be calculated by the formula $h/L=(\mathrm{\rho gL}/C_{2D})1/3$, with *L* and *ρ* denoting the length and the area density of a monolayer, respectively, and *g* represents the gravitational acceleration. Assuming the length of M_3_B_4_ to be 100 μm, we can find that the gravity-induced bending for M_3_B_4_ is lower than that of 2D CrI_3_ (6.45 × 10^−4^) [45] and CrGeTe_3_ (4.10 × 10^−4^) [46]. Therefore, our predicted 2D M_3_B_4_ demonstrates better resistance to deformation, enabling it to maintain a free-standing planar structure without the need for substrates.

To verify the experimental synthesis possibility of 2D M_3_B_4_ compounds, their cohesion energies *E*_*coh*_ were calculated by *E*_*coh* = _ (E*_M_*_*3*_*_B_*_*4*_ - 3*E_M_* - 4*E_B_*)/7, where *E*_*M3B4*_, *E*_*M*_, and *E*_*B*_ are the energies of 2D M_3_B_4_, a single transition metal atom, and a single boron atom, respectively. The *E*_*coh*_ of the five 2D M_3_B_4_ compounds was found to be between −4.11 and −5.25 eV (as shown in Table S1), which is comparable to those of the synthesized 2D materials, such as 2D silicene (−3.98 eV/atom) [47] and MoS_2_ (−5.02 eV/atom) [48].

Using 2D *h*-Mn_3_B_4_ as an example, the electron localization function (ELF) was calculated and the cross-section of calculated ELF along the (110) plane is shown in Figure 1h. According to the definition of ELF [49], the ELF values are mapping onto the range 0 ≤ ELF ≤ 1. The ELF = 1 corresponds to perfect localization, namely lone electron pair, and ELF = 0.5 is corresponding to electron gas. The values from 0.5 to 1.0 stands for the regions where there are electrons paired in covalent bonds or highly localized, unpaired electrons in dangling bonds. The ELF closing to zero indicates the fully electronic delocalization, namely no electron. In *h*-Mn_3_B_4_, the ELF between boron atoms that form a six-membered ring between the surface layer and the middle layer of M atoms is close to 1.0 (the maximum is about 0.95), indicating a strong B-B covalent bond. Conversely, there is no electron localization observed around the transition metal Mn (ELF equals 0), indicating that Mn atoms transfer electrons to the surrounding B atoms, forming Mn-B ionic bonds. Based on these observations, it can be speculated that the coexistence of B-B covalent bonds and Mn-B ionic bonds collectively enhances the thermal stability of the two-dimensional M_3_B_4_ structure. The presence of both types of bonds contributes to the overall structural integrity and stability of the 2D *h*-Mn_3_B_4_ structure. As a result of the presence of both types of bonds, the 2D *h*-Mn_3_B_4_ structure is stable and intact.

### 3.2. Magnetic properties of 2D M_3_B_4_ structures

In this part, the magnetic properties of the stable *h*-M_3_B_4_ and *ort*-M_3_B_4_ structures were investigated. Different magnetic configurations were analyzed to identify the magnetic ground state (Figures S5 and S6). For the *h*-M_3_B_4_ structures, we considered one collinear ferromagnetic (FM), three collinear antiferromagnetic (AFM), and one nonmagnetic (NM) configurations within a 2 × 2 × 1 supercell (Figure S5). As for the *ort*-M_3_B_4_ structures, we studied one collinear ferromagnetic (FM), four collinear antiferromagnetic (AFM), and one nonmagnetic (NM) configuration within a 2 × 2 × 1 supercell (Figure S6). By evaluating the variances in energy among these magnetic configurations (Table S3), it was confirmed that the magnetic ground states for *h*-Cr_3_B_4_, *h*-Mn_3_B_4_, and *h*-Fe_3_B_4_ are AFM-0, AFM-2, and AFM-2, respectively. On the other hand, the magnetic ground state for *ort*-Cr_3_B_4_ and *ort*-Mn_3_B_4_ were determined to be FM and AFM-0, respectively (Table S3).

The magnetic properties of *h*-M_3_B_4_ and *ort*-M_3_B_4_ were quantitatively investigated by calculating the magnetic coupling constant using the Heisenberg Hamiltonian (Equations (3) and (4)) [50]. Although the metallic magnetic system is described by the Stoner model in principle, it has been proved that such itinerant magnetism can be mapped onto a classic Heisenberg model with Ruderman-Kittel-Kasuya-Yosida (RKKY) exchange [51], *e.g*., metallic magnet Fe_3_GeTe_2_ [9]. Hence, it is acceptable that we employ a Heisenberg Hamiltonian model to describe these metallic systems. This approach allowed for a detailed analysis of the strength and nature of the magnetic interactions within these compounds [52].

(3) $$H_{\mathrm{spin}}=\;-J_{1}\underset{i,j}{\sum }S_{i}\cdot S_{j}-J_{2}\;\underset{i,k}{\sum }S_{i}\cdot S_{k}-J_{2\;\prime }\underset{j,m}{\sum }S_{j}\cdot S_{m}-\underset{i}{\sum }A\;\left(S_{i}^{z}\right)2-\underset{j}{\sum }A\left(S_{j}^{z}\right)2$$

(4) $$H_{\mathrm{spin}}=\;-J_{1}\underset{i,j}{\sum }S_{i}\cdot S_{j}-J_{2}\;\underset{i,k}{\sum }S_{i}\cdot S_{k}-J_{3}\;\underset{i,l}{\sum }S_{i}\cdot S_{l}-J_{2\;\prime }\;\underset{j,m}{\sum }S_{j}\cdot S_{m}-J_{3\;\prime }\;\underset{j,n}{\sum }S_{j}\cdot S_{n}-\underset{i}{\sum }A\;\left(S_{i}^{z}\right)2-\underset{j}{\sum }A\;\left(S_{j}^{z}\right)2\;$$

where *J*_1_ represents the parameter for inter-plane magnetic exchange coupling between first neighboring atoms; *J*_2_ and *J*_2’_ are the parameters that characterize the second neighboring intra-plane exchange coupling in the surface layer and middle layer, respectively; *J*_3_ and *J*_3’_ represent the parameters for third neighboring intra-plane exchange coupling in the surface layer and middle layer (arrows marked in Figure 1), respectively, within *ort*-M_3_B_4_; *S* denotes the spin vector of each M atom; *A* represents an anisotropy energy constant.

By mapping the total energies of the ferromagnetic (FM) and antiferromagnetic (AFM-i) configurations of *h*-M_3_B_4_ (where i ranged from 0 to 3) and *ort*-M_3_B_4_ (where i ranged from 0 to 4) into the Hamiltonian, a least-squares method was employed to determine the values of *J* for both compounds. The detailed results and values of *J* for *h*-M_3_B_4_ and *ort*-M_3_B_4_ can be found in Table S4. For each magnetic ground state of for *h*-M_3_B_4_ and *ort*-M_3_B_4_, calculations were performed to determine the magnetic moments and ΔQ (charge transfer from M to B atoms) within the GGA+*U* framework (Table S5). Additionally, we compared the calculated spin and orbital moments for each atom in *ort*-Cr₃B₄ as an example in Table S6. Our findings indicate that the magnetic moments in *ort*-Cr₃B₄ are primarily derived from the *d* orbitals of Cr.

**Figure 2.**
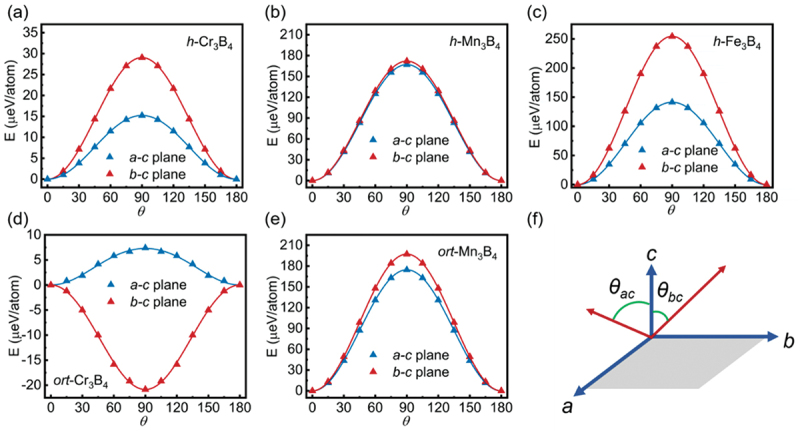
Angular dependence of the calculated magnetic anisotropy energies (*μ*eV per atom) of (a) *h*-Cr_3_B_4_, (b) *h*-Mn_3_B_4_, (c) *h*-Fe_3_B_4_, (d) *ort*-Cr_3_B_4_, and (e) *ort*-Mn_3_B_4_; (f) the illustration of spin vector *S* rotating with the polar angle *θ* through the *a – c* and *b – c* planes.

Thermal fluctuations and spin decoherence are significant challenges in quantum spin processes, including long-term information storage. A high MAE creates a substantial energy barrier that helps maintain stable and robust magnetization. This energy barrier allows the magnetization to remain oriented along a preferred spatial direction for extended periods, mitigating decoherence effects and enhancing the reliability of quantum spin processes. The magnetic anisotropy energy (MAE) quantifies the energy discrepancy between a configuration where spins are oriented in the same direction as the hard magnetization and a configuration where spins are oriented in the same direction as the easy magnetization. MAE is essential for stabilizing magnetization and preventing fluctuations in the magnetization [53]. MAE can be attributed to two main components: the magnetocrystalline anisotropy energy (C-MAE) and the magnetically dipolar anisotropy energy (D-MAE). In 2D materials, the contribution of D-MAE is generally very small and decreases with increasing layer height [54]. Therefore, our focus is primarily on C-MAE. Here, the MAE is primarily arisen from the contribution of spin-orbit coupling (SOC) [55,56]. To evaluate the MAEs of *h*-M_3_B_4_ and *ort*-M_3_B_4_, the GGA+U+SOC method, which considers the effects of SOC, was employed. The calculated MAEs are displayed in Figure 2. It shows that the M atom’s spin vector *S*(*θ*) is rotated by an angle *θ* ranging from 0° to 180° in 15° increments across the *a-c* and *b-c* planes. From Figures 2d,f, it can be observed that the hard and easy magnetization axes of *ort*-Cr_3_B_4_ appear along the *a* axis ([100]) in the *a-c* plane and the *b* axis ([010]) in the *b-c* plane, respectively. The MAE of *ort*-Cr_3_B_4_ is approximately 28.23 µeV per atom. Figures 2(a–c) and 2e demonstrate that in the *b-c* plane, the energy reaches its maximum value at 90°, indicating that the *b* axis represents the magnetic hard axis for *h*-M_3_B_4_ and *ort*-Mn_3_B_4_ (*M*  = Cr, Mn, and Fe). In the *a-c* and *b-c* planes, the energy reaches its minimum value at 0°, suggesting that *h*-M_3_B_4_ (*M*  = Cr, Mn and Fe) and *ort*-Mn_3_B_4_ are easily magnetized along the *c* axis ([001]). Moreover, by employing different k-point grid sizes from 5 × 5 × 1 to 7 × 7 × 1, we confirmed that the MAE of *ort*-Cr_3_B_4_ is converged when the *k*-point is set to 6 × 6 × 1. The easy axis of *ort*-Cr_3_B_4_ is the *b* axis with the varied *k*-point grid sizes. The calculated MAE of ort-Cr_3_B_4_ is shown in Table S7. In summary, the calculated MAEs per atom for *h*-Cr_3_B_4_, *h*-Mn_3_B_4_, *h*-Fe_3_B_4_, *ort*-Cr_3_B_4_, and *ort*-Mn_3_B_4_ are 29.05, 172.02, 255.17, 28.23, and 197.26 µeV, respectively. The values of 2D *h*-Mn_3_B_4_, *h*-Fe_3_B_4_ and *ort*-Mn_3_B_4_ exceed those of bulk Fe (1 µeV per atom) and Ni (3 µeV per atom) [57], as well as the Fe monolayer on Rh (111) (80 µeV per atom) [58], indicating that the magnetization of *h*-Mn_3_B_4_, *h*-Fe_3_B_4_ and *ort*-Mn_3_B_4_ is thermally stable. Consequently, the magnetic anisotropy energy of *h*-Mn_3_B_4_, *h*-Fe_3_B_4_ and *ort*-Mn_3_B_4_ shows potential for facilitating dense storage and quantum spin processing [59].

The equation below can be used to describe the relationship between MAE and angle *θ* [60,61]:

(5) $$E\left(\theta \right)=\;K_{1}\sin\theta 2+K_{2}\sin\theta 4$$

where *K*_1_ and *K*_2_ represent the anisotropy coefficients, *θ* denotes the polar angle measured in relation to the c axis within either the *a – c* or *b – c* plane (see Figure 2f). The *K*_1_(*K*_2_) values in the *a-c* and *b-c* planes are determined by fitting Equation (5) and are listed in Table S8. It is accurately conveyed that *K*_1_ is positive and significantly larger than *K*_2_ in the *a-c* or *b-c* planes of *h*-Cr_3_B_4_, *h*-Mn_3_B_4_, *h*-Fe_3_B_4_ and *ort*-Mn_3_B_4_. Additionally, it can be observed that *K*_1_ of *ort*-Mn_3_B_4_ in the *b-c* plane is negative. Generally, positive and dominant *K*_1_ values show a favored magnetization orientation along a vertical easy axis (*c*-axis), while negative *K*_1_ values suggest a direction perpendicular to the *c*-axis. Notably, the MAEs of *h*-Mn_3_B_4_, *h*-Fe_3_B_4_ and *ort*-Mn_3_B_4_ are significantly higher than that of some previously reported materials, such as Mn_2_NO_2_(63 µeV per atom) [36] and Cr_2_NO_2_ (22 µeV per atom) [35].

**Figure 3.**
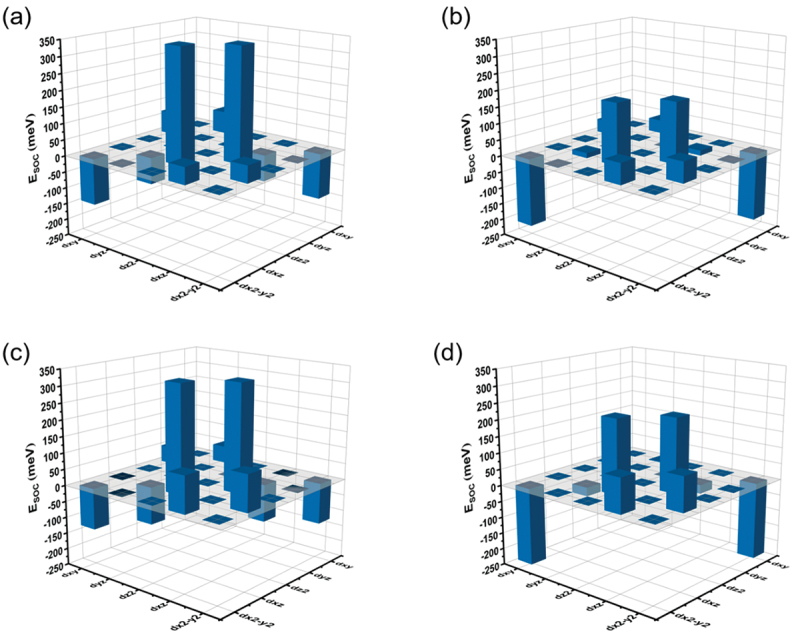
The contribution to MAEs from the SOC interaction between different *d* orbitals for M1 and M2 atoms of (a, b) *h-*Mn_3_B_4_ and (c, d) *ort-*Mn_3_B_4_.

In order to provide more clarity on the source of the magnetic anisotropy energies (MAEs) in *h*-Cr_3_B_4_, *h*-Mn_3_B_4_, *h*-Fe_3_B_4_ and *ort*-Mn_3_B_4_, we conducted a decomposition analysis of the calculated MAEs into different orbitals, as depicted in Figure 3 and Figure S8. $\mathrm{The}\;\mathrm{energy}\;\mathrm{difference}\;(\Delta E_{\mathrm{SOC}}$) caused by spin-orbit coupling (SOC) between in-plane ($\Delta E_{SOC}^{x}$) and out-of-plane ($\Delta E_{SOC}^{z}$) magnetizations is defined as $\Delta E_{\mathrm{SOC}}=\Delta E_{SOC}^{x}-\Delta E_{SOC}^{z}$. $A\;\mathrm{positive}\;\Delta E_{\mathrm{SOC}}$ indicates that the easy magnetization axis is along the *c* axis. According to the calculation results (Table S8), the $\;\Delta E_{\mathrm{SOC}}$ values of *h*-Cr_3_B_4_, *h*-Mn_3_B_4_, *h*-Fe_3_B_4_, and *ort*-Mn_3_B_4_ are positive and nearly twice as large as the MAEs, in agreement with second-order perturbation theory, i.e., MAE ≈ 1/2$\Delta E_{\mathrm{SOC}}$.

Furthermore, this suggests that the magnetic anisotropy in these four materials is primarily attributed to the contribution of SOC. Taking *h*-Mn_3_B_4_ and *ort*-Mn_3_B_4_ as examples, we observed that SOC mainly originates from the Mn atom, while the contribution from the B atom is minimal and negligible (~0.3 µeV per atom). Specifically, the SOC in *h*-Mn_3_B_4_ and *ort*-Mn_3_B_4_ is primarily derived from the *d*-orbitals of Mn atoms. From Figure 3, it can be inferred that the interaction between $d_{z2}$ and $d_{\mathrm{xz}}$ orbitals contribute the most positive SOC, with significantly higher energy than the interaction involving other *d* orbitals. The $d_{z2}$ and $d_{\mathrm{xz}}$ orbitals of Mn atoms explain the physical origin of the MAE in *h*-Mn_3_B_4_ and *ort*-Mn_3_B_4_. By comparing the *d*-orbital decomposition of Mn1 and Mn2 atoms, we find that M1 atoms contribute significantly more to the MAE than M2 atoms. The strong interaction between the$d_{\mathrm{xy}}$ and $d_{x2-y2}$ orbitals in the M2 atom is responsible to negative SOC.

**Figure 4.**
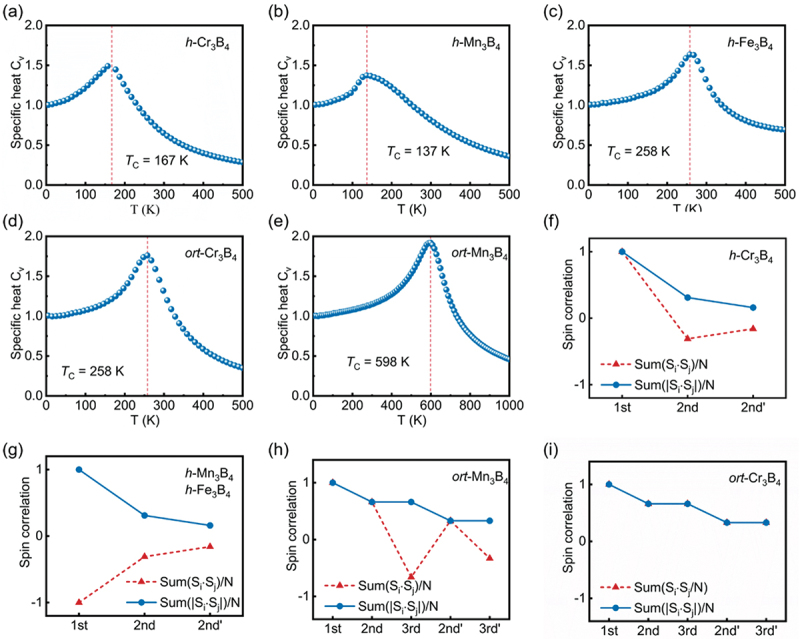
Monte carlo simulation results for 2D *h*-M_3_B_4_(*M* = Cr, Mn, Fe) and *ort*-M_3_B_4_ (*M* = Cr, Mn). Specific heat *C*_V_ was calculated as a function of temperature (*T*) for (a) *h*-Cr_3_B_4_, (b) *h*-Mn_3_B_4_, (c) *h*-Fe_3_B_4_, (d) *ort*-Cr_3_B_4_, and (e) *ort*-Mn_3_B_4_. Calculated spin-spin correlations by monte carlo simulation at *T* = 5 K for (f) *h*-Cr_3_B_4_, (g) *h*-Mn_3_B_4_, (g) *h*-Fe_3_B_4_, (h) *ort*-Cr_3_B_4_, and (i) *ort*-Mn_3_B_4_.

Through the comparison of the energies of various magnetic configurations, the magnetic ground states of these MBenes at low temperature (*T* = 5 K) [62] were identified through calculating the spin-spin correlations during Monte Carlo (MC) simulations. An average value of the products of neighboring spins $\mathrm{Sum}\left(S_{i}\cdot S_{j}\right)/N$ and their absolute values$\mathrm{Sum}\left(\left|S_{i}\cdot S_{j}\right|\right)/N$ for the spin Hamiltonian with the given coupling constants is shown in Figure 4. The parameter N denotes the dimensions of the lattice used in the MC simulation. Figures 4 (f) and (g) demonstrate that the calculated values of $\mathrm{Sum}\left(S_{i}\cdot S_{j}\right)/N$ for 2D *h*-Cr_3_B_4_, *h*-Mn_3_B_4_ and *h*- Fe_3_B_4_ are 1, −1 and −1, respectively, for the 1^st^ neighboring spins (inter-plane spins), indicating FM coupling for *h*-Cr_3_B_4_ and AFM coupling for *h*-Mn_3_B_4_ and *h*-Fe_3_B_4_. The 2^nd^ nearest neighboring spins $\mathrm{Sum}\left(S_{i}\cdot S_{j}\right)/N$ of *h*-M_3_B_4_ (*M* = Cr, Mn, Fe) in the surface layer and middle layer are −0.33 and −0.16, respectively, indicating the presence of both FM and AFM couplings that do not completely cancel out for the 2^nd^ spin coupling in the surface layer and middle layer, resulting in the proposed AFM-0 and AFM-2 configurations. Figures 4 (h) and (i) show that the calculated $\mathrm{Sum}\left(S_{i}\cdot S_{j}\right)/N$ for the 1^st^ nearest neighboring spin is 1 for *ort*-Cr_3_B_4_ and *ort*-Mn_3_B_4_, indicating a FM coupling is apparent between M1 atoms in the surface and M2 atoms in the middle layer. The 2^nd^ and 3^rd^ nearest neighbor spins $\mathrm{Sum}\left(S_{i}\cdot S_{j}\right)/N$ of *ort*-Cr_3_B_4_ are 0.66 in the surface layers and 0.33 in the middle layers, which indicates magnetic coupling of the 2^nd^ and 3^rd^ nearest neighbor spins in both the surface and middle layers of *ort*-Cr_3_B_4_. The calculation results of the spin correlation coefficients of the *ort*-Cr_3_B_4_ confirm that FM is its magnetic ground state.

The 2^nd^ nearest neighbor spins $\mathrm{Sum}\left(S_{i}\cdot S_{j}\right)/N$ in *ort*-Mn_3_B_4_ are 0.66 and 0.33 in the surface and middle layers, respectively. This suggests that magnetic coupling of the 2^nd^ neighbor spins is FM in both the surface and middle layers. On the other hand, the 3^rd^ nearest neighbor spins $\mathrm{Sum}\left(S_{i}\cdot S_{j}\right)/N$ in *ort*-Mn_3_B_4_ are −0.66 and −0.33 in the surface and middle layers, respectively, indicating that the magnetic coupling of the 3^rd^ neighbor spins is AFM in both the surface and middle layers. The Monte Carlo simulation results further confirm that the AFM-0 configuration is the ground state of *ort*-Mn_3_B_4_.

The Curie temperature (*T*_*C*_) and Néel temperature (*T*_*N*_) are critical temperatures that respectively indicate the transition of ferromagnetic and antiferromagnetic materials to paramagnetic materials. These temperatures are important parameters for spintronic device applications. From Figures 4(a–e), it can be observed that the Monte Carlo simulations yielded critical temperatures of 167 K, 137 K, 258 K, 258 K, and 598 K for *h*-Cr_3_B_4_, *h*-Mn_3_B_4_, *h*-Fe_3_B_4_, *ort*-Cr_3_B_4_, and *ort*-Mn_3_B_4_, respectively. Additionally, applying the same method, the Curie temperature (*T*_*C*_) of CrI_3_ monolayer was calculated as 53 K (Figure S7), which is a close match to the theoretical value of 51 K [63,64]. This demonstrates the accuracy of the calculation method employed in this study.

### 3.3. The origin of magnetism in M_3_B_4_ MBenes

To investigate the underlying cause of magnetism in the most stable magnetic configurations of 2D *h*-M_3_B_4_ (*M* = Cr, Mn, Fe) and *ort*-M_3_B_4_ (*M* = Cr, Mn), their electronic structures were computed using the GGA+*U* functional. As depicted in Figure S10, the calculations reveal that these materials exhibit metallic behavior, with energy bands of the transition metal atoms crossing the Fermi level. This characteristic endows *h*-M_3_B_4_ and *ort*-M_3_B_4_ with high conductivity. Moreover, taking *ort*-Cr_3_B_4_ as an example, we plotted its band structures and density of states by employing the different *U*_*eff*_ values. As shown in Figure S11, its metallic behavior is preserved across the range of *U*_*eff*_ values from 2 eV to 4 eV, with only minor changes in the band structure. There calculations herein confirmed the rationality of *U*_*eff*_ = 2 eV.

**Figure 5.**
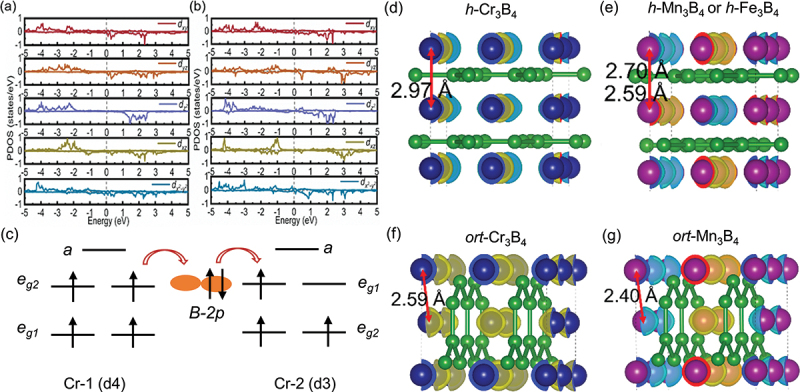
Projected density of state (PDOS) for (a) M1 and (b) M2 atoms of of $d_{\mathrm{xy}}$, $d_{\mathrm{yz}}$, $d_{z2}$, $d_{\mathrm{xz}}$ and $d_{x2-y2}$ of 2-D *h*-Mn_3_B_4_. The calculation is done under GGA+*U* (*U* = 2 eV) functional. (c) Schematic diagram illustrating the double exchange interaction between *h*-Cr_3_B_4_ layers. *a* corresponds to $d_{z2}$, *e*_*g1*_ and *e*_*g2*_ stand for $(d_{\mathrm{xy}}$, $d_{x2-y2}$) and ($d_{\mathrm{yz}}$, $d_{\mathrm{xz}}$), respectively. Side views of spin-charge densities for the magnetic ground states of (d) *h*-Cr_3_B_4_, (e) *h*-Mn_3_B_4_ or *h*-Fe_3_B_4_, (f) *ort*-Cr_3_B_4_, and (g) *ort*-Mn_3_B_4_.

Density of state (DOS) calculations can reveal interesting characteristics of the materials. The calculated DOS of 2D *ort*-Cr_3_B_4_ always exhibits asymmetry when the U_eff_ varies from 2.0 eV to 4.0 eV, while the DOS of other materials is completely symmetric. This asymmetry suggests that *ort*-Cr_3_B_4_ is FM, while the other materials are AFM. The M atom is the main contributor to the material’s magnetic properties, as observed from the contribution of atomic components. The DOS of M atoms overlaps with that of B atoms, forming an M-B ionic bond. Furthermore, the origin of magnetic moments in 2D *h*-M_3_B_4_ and *ort*-M_3_B_4_ is analyzed, and the projected state density (PDOS) of *d* orbitals of M1 and M2 atoms were calculated and are plotted in (Figure 5 a and b) and Figure S12. By observing the shape similarity of PDOS, we can deduce that the crystal field of *h*-M_3_B_4_ causes the five degenerate 3*d* orbitals of M to split into singlet states *a* ($d_{z2}$), *e*_*g1*_ ($d_{\mathrm{xy}}$, $d_{x2-y2}$) and *e*_*g2*_ ($d_{\mathrm{yz}}$, $d_{\mathrm{xz}}$).

To further understand the magnetic coupling interaction, we calculated the band center (*ε*_d_) and occupation number (*m*_*d*_) of the partial *d* orbitals of M in *h*-Cr_3_B_4_ considering its intrinsic metallic magnetism (Figure S13). The *ε*_d_ and *m*_*d*_ can be obtained using the below equations respectively [20,65]:

(6) $$\varepsilon_{d}\;=\;\int_{-\infty }^{\infty }n_{d}\left(\varepsilon \right)\mathrm{\varepsilon d\varepsilon}/\int_{-\infty }^{\infty }n_{d}\left(\varepsilon \right)\mathrm{d\varepsilon}$$

(7) $$m_{d}\;=\;\int_{-\infty }^{E_{f}}n_{d}\left(\varepsilon \right)\mathrm{d\varepsilon}$$

where *n*_*d*_*(ε)* is corresponding to the DOS of the partial d orbitals of an M atom in M_3_B_4_ at a given energy *ε* and *E*_*f*_ is the Fermi level that is set to zero. By comparing the energy differences between different *d* orbitals, we can find that the order of energy level is *e*_*g1*_ ($d_{\mathrm{xy}}$, $d_{x2-y2})$, *e*_*g2*_ ($d_{\mathrm{yz}}$, $d_{\mathrm{xz}}$), and *a* ($d_{z2}$) for Cr-1 while that is *e*_*g2*_ ($d_{\mathrm{yz}}$, $d_{\mathrm{xz}}$), *e*_*g1*_ ($d_{\mathrm{xy}}$, $d_{x2-y2})$, and *a* ($d_{z2}$) for Cr-2. These orders are consistent from the triangle planar crystal field and two types of Cr atoms with different coordinated numbers. By counting the occupation number (*m*_*d*_) of partial *d* orbitals, we obtained that the *m*_*d*_ of Cr-1 and Cr-2 in the spin-up state is 3.91 and 3.43 while that of Cr-1 and Cr-2 in the spin-down state is 0.46 and 1.08. The total occupation numbers of Cr-1 and Cr-2 are 3.45 and 2.35, close to the magnetic moment of Cr-1 and Cr-2 in *h*-Cr_3_B_4_ (3.55 µB and 2.49 µB).

Figures 5d, e illustrate the side views of the spin-charge densities for the magnetic ground states of *h*-Cr_3_B_4_ and *h*-M_3_B_4_ (*M* = Mn, Fe), respectively. It is observed that *h*-Cr_3_B_4_ exhibits interlayer FM coupling, while *h*-Mn_3_B_4_ and *h*-Fe_3_B_4_ exhibit AFM coupling. The reason for this difference can be attributed to the distance between M1 and M2 in *h*-Cr_3_B_4_, which is larger (2.97 Å) compared to the distance between M1 and M2 in *h*-M_3_B_4_ (2.70 Å for *h*-Mn_3_B_4_ and 2.59Å for *h*-Fe_3_B_4_). It is well known that direct exchange interactions between transition metal atoms typically favor AFM magnetic ordering. Therefore, the dominant direct exchange interaction in *h*-M_3_B_4_ (*M* = Mn, Fe) leads to the observed interlayer AFM coupling in comparison with *h*-Cr_3_B_4_ with FM interlayer coupling. The M1 and M2 atoms in *h*-M_3_B_4_ (*M* = Mn, Fe) have different numbers of neighboring B atoms, resulting in different electron transfers from M1 and M2 to surrounding B atoms, as shown in Table S5. Taking *h*-C_3_B_4_ as an example, it can be inferred that the Cr1 and Cr2 atoms in *h*-Cr_3_B_4_ have different local coordination environments and exhibit different valence states as Cr^2+^ and Cr^3+^, respectively. According to crystal field theory and Hund’s rule, four *d* electrons of Cr-1 fully occupy the lower energy *e*_*g1*_ and *e*_*g2*_ states. Similarly, the three *d* electrons of Cr-2 occupy the *e*_*g*1_ and *e*_*g2*_ states but the *e*_*g1*_ is partially occupied. Due to the strong Hund coupling, the *e*_*g*1_ electron transition needs to overcome a large Coulomb potential if the spins of two adjacent ions are antiparallel, making the transition forbidden. As shown in Figure 5c, when the spins of the two ions are aligned in parallel, the electrons are transferred to other ions via the 2*p* orbital of the B atom. This electron transition results in the ferromagnetic exchange of local spins in interlayers of *h*-Cr_3_B_4_. Therefore, in addition to direct exchange interactions, double exchange interactions also play a role in the 2D *h*-M_3_B_4_ magnets. Double exchange occurs between ions with distinct oxidation states, where one atom has an extra electron compared to the other. The coordinated B ions in 2D *h*-M_3_B_4_ have negligible magnetic moments (Figure 5d, e).

Finally, it is worth mentioning that the *ort*-M_3_B_4_ structure bears resemblance to *ort*-MB [20]. Upon comparison, it is observed that *ort*-M_3_B_4_ and *ort*-M_2_B_2_ (*M* = Cr, Mn) exhibit similar magnetic ground states. Additionally, in *ort*-M_3_B_4_ (*M*  = Cr, Mn), M1 and M2 are in different oxidation states due to the varying number of electrons they transfer to the surrounding B atoms (Table S5). Furthermore, influenced by double exchange, the inter-layers of *ort*-Cr_3_B_4_ and *ort*-Mn_3_B_4_ are coupled by ferromagnetic (FM) interaction (see Figures 5f, g).

## 4. Conclusions

In this work, we presented compelling evidence regarding the structural, electronic, and magnetic properties of *h*-M_3_B_4_ and *ort*-M_3_B_4_ MBenes. Our findings indicate that both 2D *h*-M_3_B_4_ and *ort*-M_3_B_4_ exhibit remarkable stability and intriguing magnetic characteristics. Compared to common magnetic 2D materials, M_3_B_4_ exhibits a significantly higher in-plane elastic modulus and a relatively smaller deformation under the influence of gravity in the out-of-plane direction. This characteristic enables M_3_B_4_ to better retain its exceptional magnetic properties even when subjected to significant changes in the external environment. As for its magnetic properties, 2D *ort*-Cr_3_B_4_ is identified as a FM metal, whereas *h*-M_3_B_4_ (*M*  = Cr, Mn, and Fe) and *ort*-Mn_3_B_4_ are classified as AFM metals. Through non-collinear magnetic calculations incorporating spin–orbit coupling, we have discovered that 2D *ort*-Cr_3_B_4_ possesses an in-plane magnetic easy axis, accompanied by a magnetic anisotropy energy of 28.23 μeV per formula unit (f.u.). On the other hand, Ising antiferromagnets such as 2D *h*-M_3_B_4_ (*M* = Cr, Mn, Fe), and *ort*-Mn_3_B_4_ have out-of-plane easy magnetic axes and robust magnetic anisotropy energies of 29.05, 172.02, 254.16, and 197.26 μeV per formula unit (f.u.). Moreover, we successfully confirmed the magnetic ground states of *h*-M_3_B_4_ (*M* = Cr, Mn, Fe) and *ort*-Mn_3_B_4_, and have calculated their corresponding critical temperatures. Specifically, the critical temperatures for 2D *h*-Cr_3_B_4_, *h*-Mn_3_B_4_, *h*-Fe_3_B_4_, *ort*-Cr_3_B_4_, and *ort*-Mn_3_B_4_ are determined to be 167 K, 137 K, 258 K, 258 K, and 598 K, respectively. Our theoretical analysis revealed that interaction between the M1 (surface layer) and M2 (middle layer) transition metals is crucial for the magnetic properties of M_3_B_4_ MBenes. First, the direct exchange interaction between M1 and M2 atoms is responsible for the AFM coupling between interlayers of *h*-M_3_B_4_ (*M* = Mn, Fe). Furthermore, the interlayer FM coupling observed in *h*-Cr_3_B_4_ and *ort*-M_3_B_4_s (*M* = Cr, Mn) arises from the double exchange mechanism between M1 and M2 atoms. Furthermore, these results contribute to the broader understanding of 2D materials with potential applications in various fields, along with providing new insight into the magnetic properties of *h*-M_3_B_4_ and *ort*-M_3_B_4_ MBenes.

## Supplementary Material

Supplemental Material

## Data Availability

Data will be made available on request.
