# Supplementary material for: Theoretical investigations of two-dimensional intrinsic magnets derived from transition-metal borides M3B4 (M = Cr, Mn, and Fe)
Source: Sci Technol Adv Mater. 2024 Oct 9;25(1):2404384. doi: 10.1080/14686996.2024.2404384 (PMC11573340; doi:10.1080/14686996.2024.2404384)
Supplement: Supplemental Material [file TSTA_A_2404384_SM0829.docx]

Supplementary Materials

**Theoretical investigations of two-dimensional intrinsic magnets derived from transition-metal borides M_3_B_4_ (M = Cr, Mn, and Fe)**

Chunmei Ma^a, b#^, Shiyao Wang^a, b#^, Chenguang Gao^b^ and Junjie Wang*^a, b^

^a^ State Key Laboratory of Solidification Processing, Northwestern Polytechnical University, Xi’an, China.

^b^ School of Materials Science and Engineering, Northwestern Polytechnical University, Xi’an, China.

Corresponding author: Junjie Wang

State Key Laboratory of Solidification Processing, School of Materials Science and Engineering, Northwestern Polytechnical University, Xi’an, 710072, China.

E-mail: wang.junjie@nwpu.edu.cn


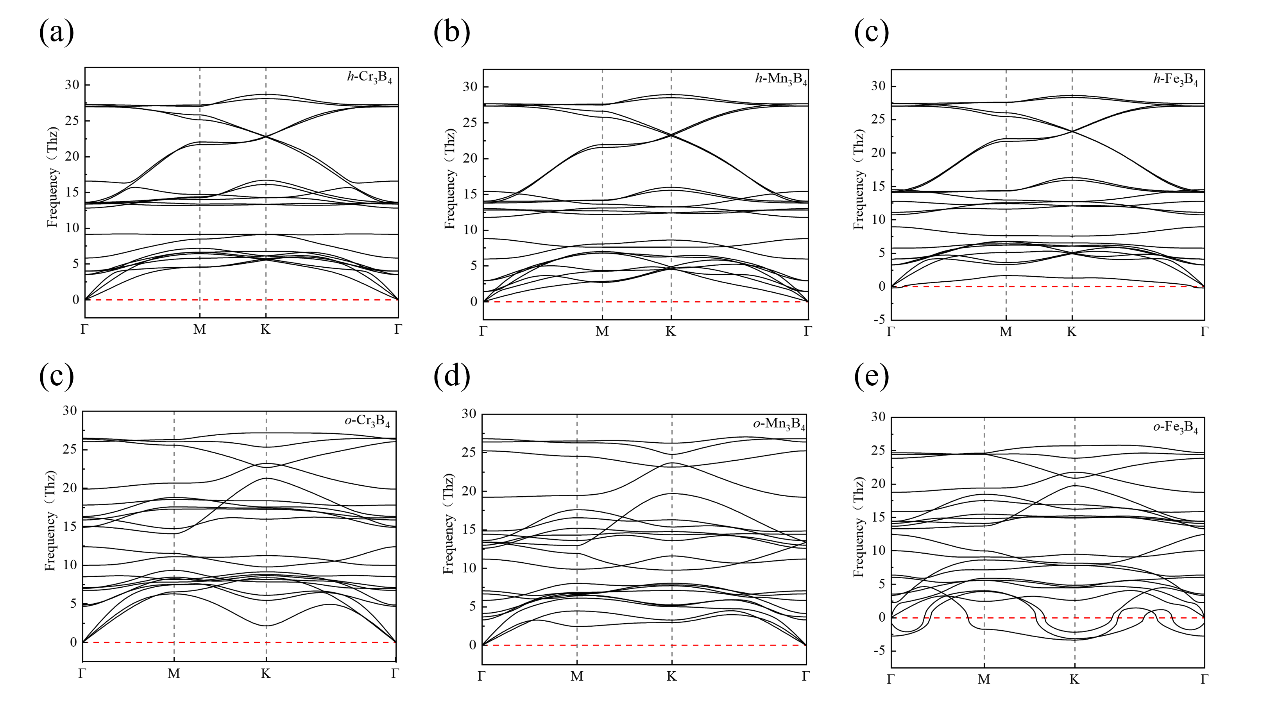


(d)

(e)

(f)

Figure S1. Calculated phonon spectra of 2D *h*-Cr_3_B_4_ (a), *h*-Mn_3_B_4_ (b), *h*-Fe_3_B_4_ (c), *ort*-Cr_3_B_4_ (d), *ort*-Mn_3_B_4_ (e), and *ort*-Fe_3_B_4_ (f).

Figure S2. Snapshots of 2D *h*-Cr_3_B_4_ (a), *h*-Mn_3_B_4_ (b), *h*-Fe_3_B_4_ (c), *ort*-Cr_3_B_4_ (d), and *ort*-Mn_3_B_4_ (e) after 11 ps AIMD simulations at 600 K.


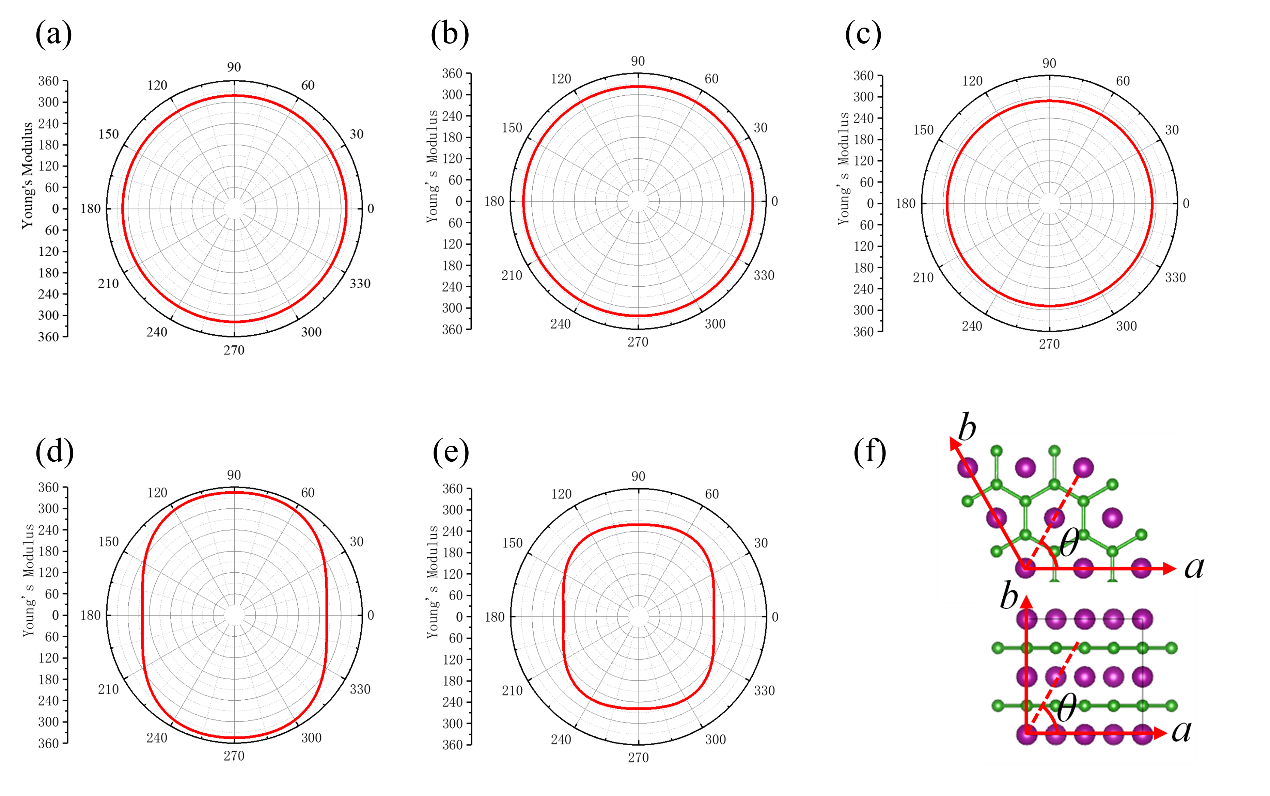


Figure S3. Young’s modulus of *h*-Cr_3_B_4_ (a), *h*-Mn_3_B_4_ (b), *h*-Fe_3_B_4_ (c), *ort*-Cr_3_B_4_ (d), and *ort*-Mn_3_B_4_ (e) as a function of the angle θ. (f) θ = 0◦ corresponds to the *a* axis.


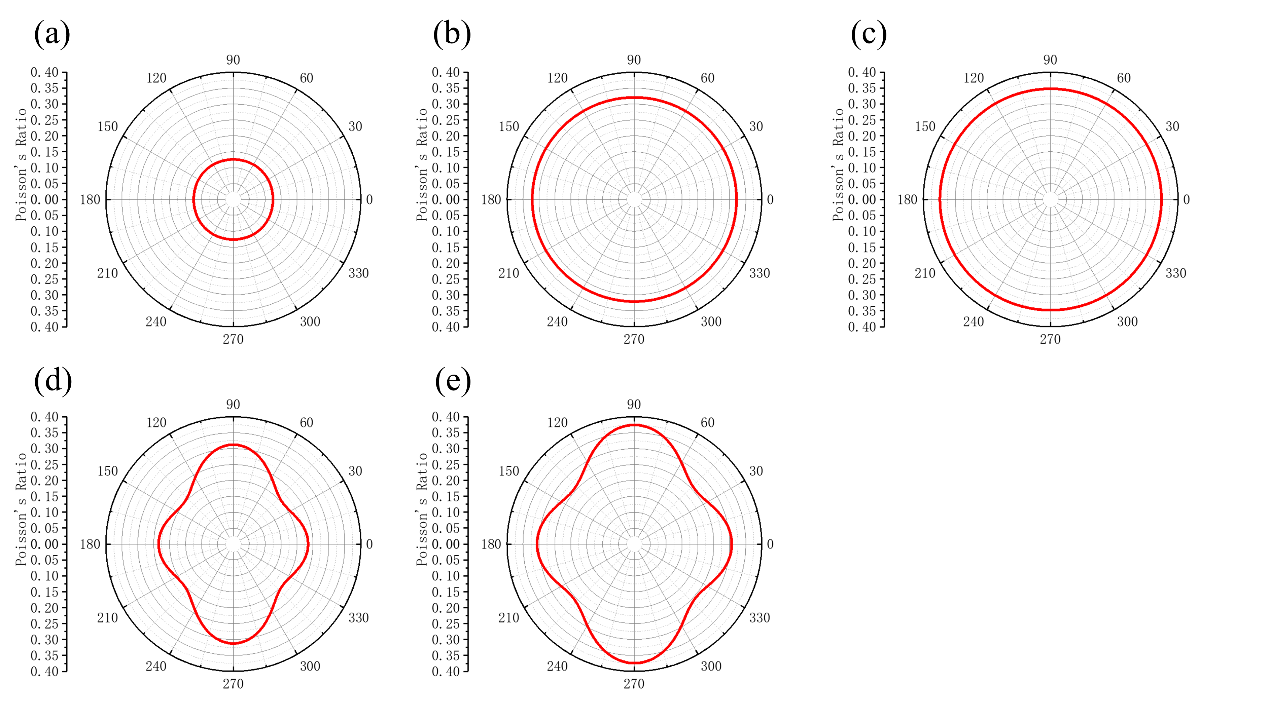


Figure S4. Poisson’s ratio of *h*-Cr_3_B_4_ (a), *h*-Mn_3_B_4_ (b), *h*-Fe_3_B_4_ (c), *ort*-Cr_3_B_4_ (d), and *ort*-Mn_3_B_4_ (e) as a function of the angle θ.

**
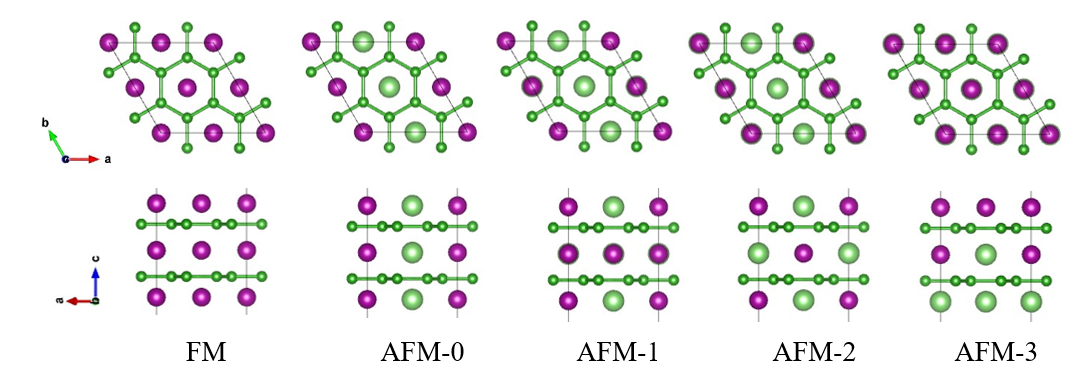
**

Figure S5. Top and side view of five collinear magnetic configurations of a 2D *h*-M_3_B_4_. FM and AFM-i (i = 0, 1, 2, and 3) respectively means ferromagnetic and antiferromagnetic state.


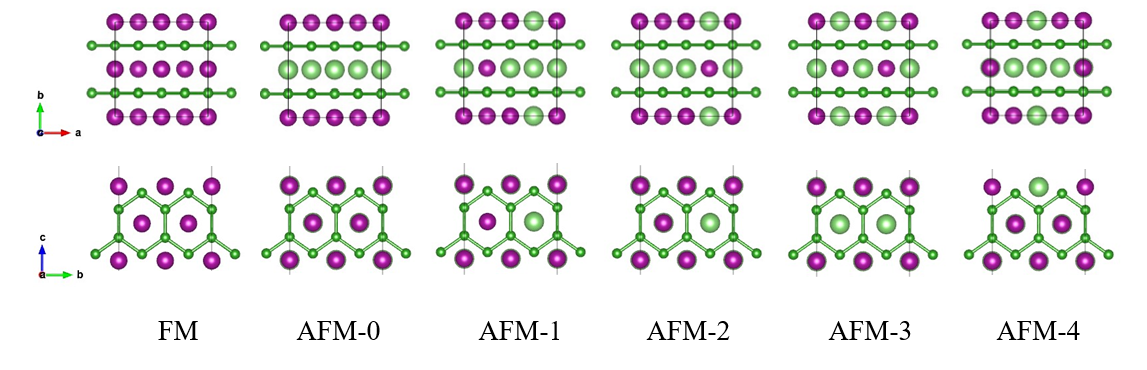


Figure S6. Top and side view of five collinear magnetic configurations of a 2D *ort*-M_3_B_4_. FM and AFM-i (i = 0, 1, 2, 3, and 4) respectively means ferromagnetic and antiferromagnetic state.

Figure S7. Simulated normalized |*S*| (black data) and specific heat *C_V_* as a function of temperature for monolayer CrI_3_.

Figure S8. The contribution to MAE from the SOC interaction between different *d* orbitals for M1, M2 atoms of *h*-Cr_3_B_4_ (a, b), *a-c* and *b-c* plane of *ort*-Cr_3_B_4_ (c, d, e, f).

Figure S8. The contribution to MAE from the SOC interaction between different *d* orbitals for M1, M2 atoms of *ort*-Mn_3_B_4_ (g, h) and *h*-Fe_3_B_4_ (i, j).


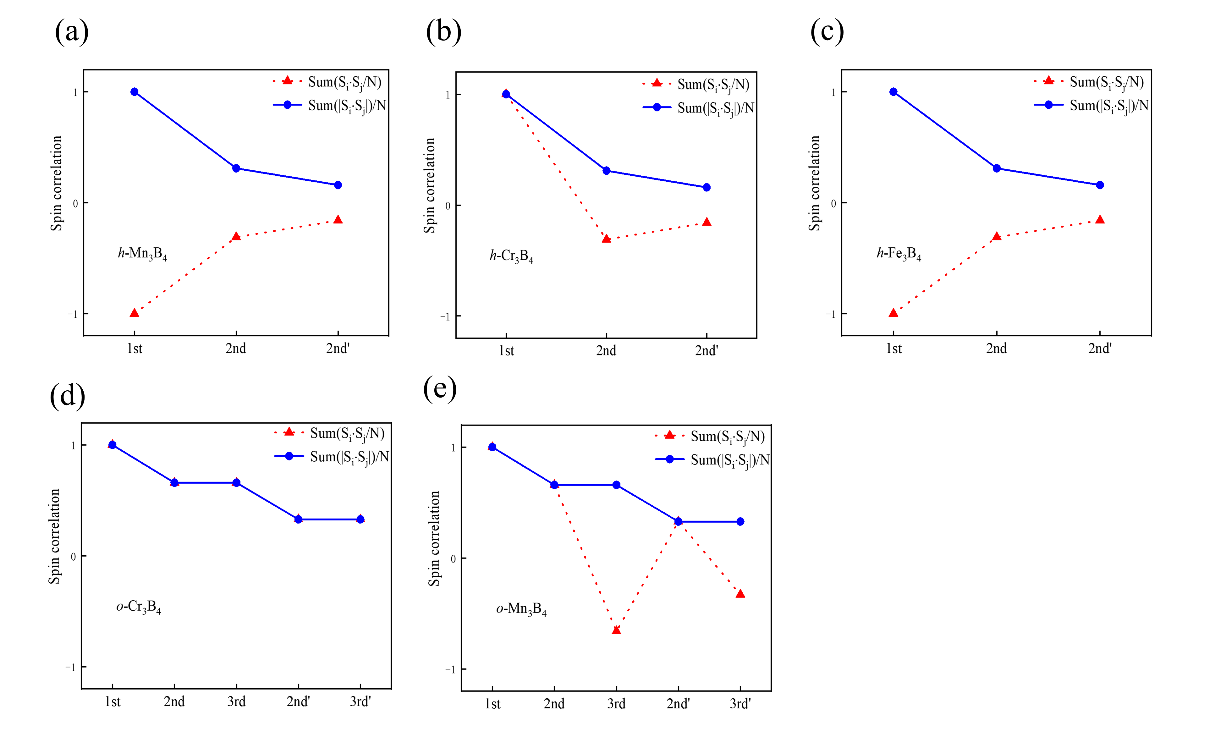


Figure S9. Calculated spin–spin correlations by Monte Carlo simulation at T = 5 K for *h*-Cr_3_B_4_ (a), *h*-Mn_3_B_4_ (b), *h*-Fe_3_B_4_ (c), *ort*-Cr_3_B_4_ (d), and *ort*-Mn_3_B_4_ (e).


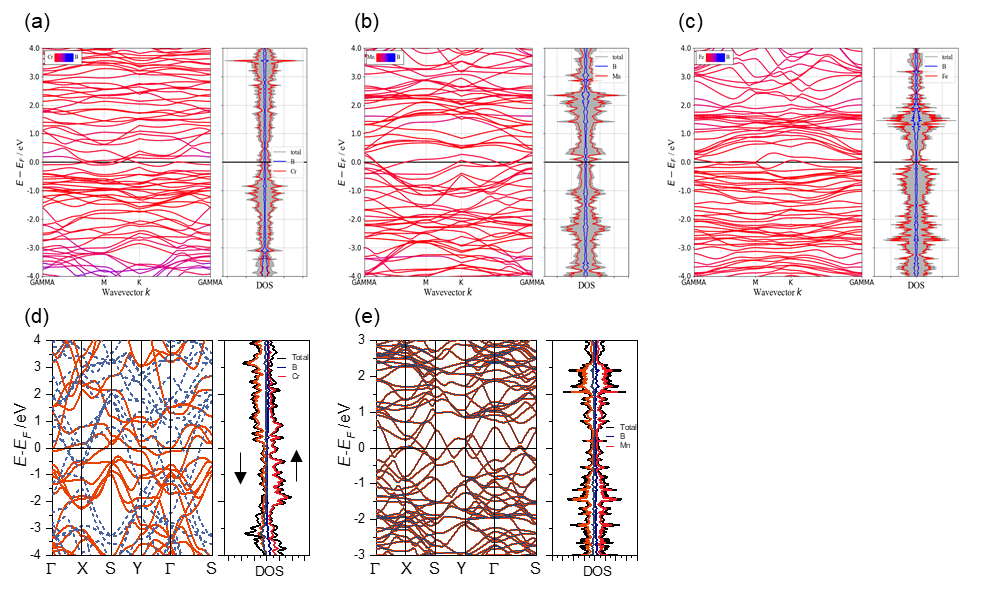


Figure S10. Calculated band structure of 2D (a) *h*-Cr_3_B_4_, (b) *h*-Mn_3_B_4_, (c) *h*-Fe_3_B_4_, (d) *ort*-Cr_3_B_4_ and (e) *ort*-Mn_3_B_4_ in their magnetic ground state by employing GGA+U (U_eff_ = 2.0 eV). The solid and dash lines respectively mean spin-up and spin-down channel. The contribution of DOS for each 2D M_3_B_4_s around the Fermi level are from transition-metal M atoms.


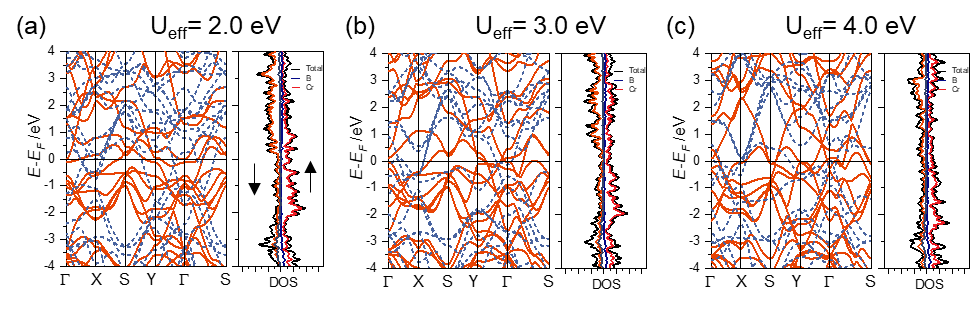


Figure S11. Calculated band structure of 2D *ort*-Cr_3_B_4_ in their magnetic ground state by employing different GGA+U (a) U_eff_ = 2.0 eV, (b) U_eff_ = 3.0 eV and (c) U_eff_ = 4.0 eV. The solid and dash lines respectively mean spin-up and spin-down channel.

Figure. S12 Projected density of state (PDOS) for M1 and M2 atoms of $d_{xy}$ ${d_{yz} d}_{z^{2}}d_{xz}$ and $d_{x^{2}-y^{2}}$ of 2D (a, b) *h*-Cr_3_B_4_, (c, d) *h*-Mn_3_B_4_ using GGA+U. (U_eff_ =2.0 eV). The Fermi levels are set to zero.

Figure. S12 Projected density of state (PDOS) for M1 and M2 atoms of $d_{xy}$ ${d_{yz} d}_{z^{2}}d_{xz}$ and $d_{x^{2}-y^{2}}$ of 2D (e, f) *h*-Fe_3_B_4_, (g, h) *ort*-Cr_3_B_4_, and (i, j) *ort*-Mn_3_B_4_ using GGA+U. (U_eff_ =2.0 eV). The Fermi levels are set to zero.


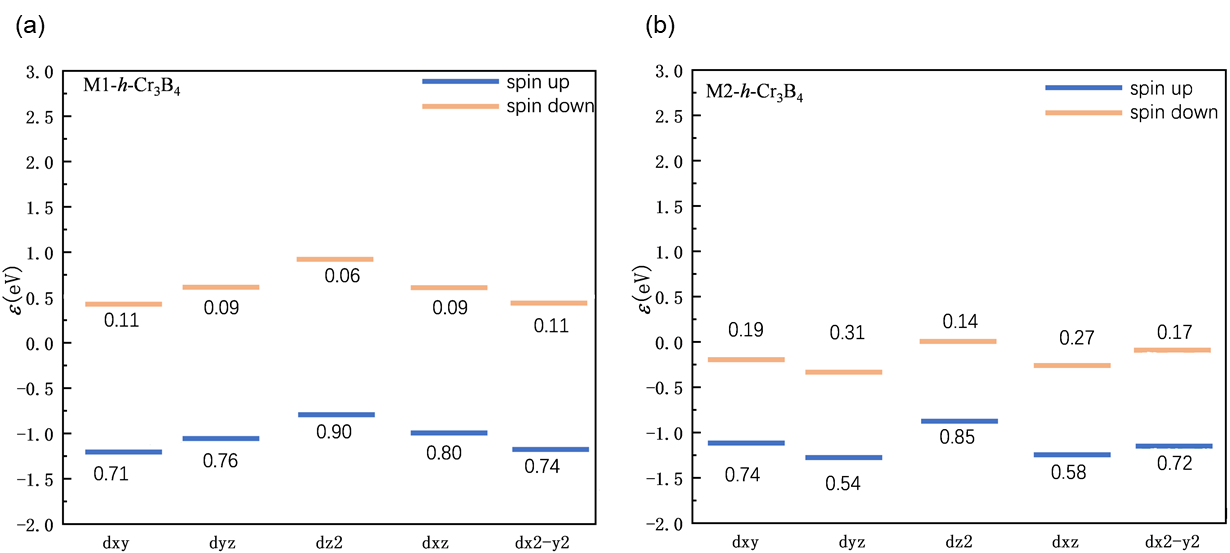


Figure. S13 Energy (*ε*) diagrams of the calculated band centers of partial *d* orbitals for the (a) M-1 and (b) M-2 in the *h*-Cr_3_B_4_ under the GGA+U (U_eff_ = 2.0 eV) functional. The number stands for the occupation of spin-up and spin-down orbitals. Orange and blue short bars donate the band center with spin-up and spin-down.

Table S1. a and b (in Å) are lattice constant. *h* (in Å) is layer height. *l*_1_, *l*_2_, *l*_3_, *l*_4_ (in Å) are bond length between M1 and B, M2 and B atom. *E_coh_* (in eV) is the cohesion energies.

| 2D | *a* | *b* | *h* | *l*_1_ | *l*_2_ | *l*_3_ | *l*_4_ | *E_coh_* |
| --- | --- | --- | --- | --- | --- | --- | --- | --- |
| *h*-Cr_3_B_4_ | 2.952 | 2.952 | 5.546 | 2.119 | / | 2.279 | / | -4.11 |
| *h*-Mn_3_B_4_ | 3.006 | 3.006 | 5.417 | 2.117 | / | 2.292 | / | -4.73 |
| *h*-Fe_3_B_4_ | 2.999 | 2.999 | 5.175 | 2.075 | / | 2.255 | / | -5.52 |
| *ort*-Cr_3_B_4_ | 2.934 | 2.915 | 4.709 | 2.126 | 2.083 | 2.246 | 2.367 | -4.56 |
| *ort*-Mn_3_B_4_ | 2.905 | 2.881 | 4.551 | 2.072 | 2.022 | 2.222 | 2.426 | -5.21 |
| *ort*-Fe_3_B_4_ | 2.837 | 2.913 | 4.642 | 2.072 | 2.021 | 2.215 | 2.387 | / |

Table S2. Elastic properties of the 2D *h*-M_2_B_2_ and *ort*-M_2_B_2_, including elastic constants *C*_ij_ (in N m^−1^), Young's modulus Y_x_, Y_y_ (in N m^−1^), and Poisson's ratio $\vartheta$_x_, $\vartheta$_y_ (dimensionless) along the x, y directions.

|  | *C*_11_ | *C*_12_ | *C*_22_ | *C*_66_ | *Y_x_* | *Y_y_* | $\vartheta$_x_ | $\vartheta$_y_ | *h/l* |
| --- | --- | --- | --- | --- | --- | --- | --- | --- | --- |
| *h*-Cr_2_B_2_ | 218.38 | 31.64 | 217.05 | 92.43 | 213.77 | 213.77 | 0.14 | 0.14 | 2.33*10^-4^ |
| *ort*-Cr_2_B_2_ | 151.84 | 57.76 | 205.34 | 77.96 | 135.59 | 183.39 | 0.28 | 0.38 | 2.41*10^-4^ |
| *h*-Mn_2_B_2_ | 164.65 | 29.58 | 164.65 | 67.54 | 159.34 | 159.34 | 0.18 | 0.18 | 2.57*10^-4^ |
| *ort*-Mn_2_B_2_ | 157.61 | 71.17 | 179.90 | 80.45 | 129.46 | 147.76 | 0.40 | 0.45 | 2.49*10^-4^ |
| *h*-Fe_2_B_2_ | 113.04 | 13.03 | 113.04 | 63.03 | 111.53 | 111.53 | 0.12 | 0.12 | 2.97*10^-4^ |

Table S3. E_FM_ and E_AFM-i_ (in eV) are total energies of FM, AFM-i, and NM by using GGA+U (i = 0, 1, 2,3 for *h*-M_3_B_4_, i = 0, 1, 2, 3, 4 for *ort*-M_3_B_4_).

| U = 2 eV | E_FM_ | E_AFM-0_ | E_AFM-1_ | E_AFM-2_ | E_AFM-3_ | E_AFM-4_ | E_NM_ |
| --- | --- | --- | --- | --- | --- | --- | --- |
| *h*-Cr_3_B_4_ | -198.222 | **-199.821** | -199.720 | -199.594 | -199.049 | / | -187.674 |
| *h*-Mn_3_B_4_ | -191.423 | -192.753 | -193.304 | **-193.403** | -192.651 | / | -175.643 |
| *h*-Fe_3_B_4_ | -181.199 | -182.312 | -182.962 | **-183.797** | -182.896 | / | -168.767 |
| *ort*-Cr_3_B_4_ | **-197.779** | -196.808 | -196.874 | -196.720 | -196.270 | -197.703 | -186.451 |
| *ort*-Mn_3_B_4_ | -194.545 | **-196.325** | -195.012 | -194.093 | -194.382 | -195.267 | -177.489 |
| U= 3 eV | E_FM_ | E_AFM-0_ | E_AFM-1_ | E_AFM-2_ | E_AFM-3_ | E_AFM-4_ | E_NM_ |
| *h*-Cr_3_B_4_ | -187.471 | **-188.725** | -188.632 | -188.561 | -187.938 | / | -177.564 |
| *h*-Mn_3_B_4_ | -182.824 | -184.059 | -184.397 | **-184.747** | -183.830 | / | -165.462 |
| *h*-Fe_3_B_4_ | -171.443 | -170.512 | -171.594 | **-172.209** | -171.034 | / | -158.425 |
| *ort*-Cr_3_B_4_ | **-184.820** | -183.903 | -183.991 | -183.812 | -183.341 | -184.783 | -166.685 |
| *ort*-Mn_3_B_4_ | -185.707 | **-187.780** | -185.320 | -186.013 | -185.871 | -185.342 | -167.378 |
| U= 4 eV | E_FM_ | E_AFM-0_ | E_AFM-1_ | E_AFM-2_ | E_AFM-3_ | E_AFM-4_ | E_NM_ |
| *h*-Cr_3_B_4_ | -178.589 | **-179.984** | -179.875 | -179.769 | -179.119 | / | -177.895 |
| *h*-Mn_3_B_4_ | -175.030 | -176.697 | -177.011 | **-177.306** | -175.914 | / | -155.661 |
| *h*-Fe_3_B_4_ | -162.348 | -162.129 | -162.865 | **-163.544** | -162.471 | / | -148.767 |
| *ort*-Cr_3_B_4_ | **-175.421** | -174.350 | -174.426 | -174.295 | -173.890 | -175.333 | -155.445 |
| *ort*-Mn_3_B_4_ | -175.338 | **-177.780** | -175.829 | -175.9069 | -175.117 | -176.340 | -157.343 |

Table S4. Magnetic couple constants *J*_1_, *J*_2_, *J*_2’_ and T_c_ of *h*-M_3_B_4_, and magnetic couple constants *J*_1_, *J*_2_, *J*_3_, *J*_2’_, *J*_3’_ and T_c_ of *ort*-M_3_B_4_ by using GGA+U.

|  | *J*_1_(meV) | *J*_2_(meV) | *J*_2_’(meV) | *J*_3_(meV) | *J*_3_’(meV) | T_c_(K) |
| --- | --- | --- | --- | --- | --- | --- |
| *h*-Cr_3_B_4_ | 14.2 | -20.7 | -58.8 | / | / | 168 |
| *h*-Mn_3_B_4_ | -38.1 | -34.1 | -36.2 | / | / | 137 |
| *h*-Fe_3_B_4_ | -66.8 | -7.4 | 38.5 | / | / | 258 |
| *ort*-Cr_3_B_4_ | 16.8 | -77.7 | -22.7 | 51.1 | 19.2 | 258 |
| *ort*-Mn_3_B_4_ | 81.4 | -52.8 | 64.1 | -144.1 | -9.7 | 598 |

*E_FM_ = E*_0_ – 8*J*_1_*|S|*^2^ – 24*J*_2_*|S|*^2^ – 12*J*_2’_ *|S|*^2^ – *A|S|*^2^

*E_AFM-_*_0_ *= E*_0_ – 8*J*_1_*|S|*^2^ + 8*J*_2_*|S|*^2^ + 4*J*_2’_ *|S|*^2^ – *A|S|*^2^

*E_AFM-_*_1_ *= E*_0_ – 0*J*_1_*|S|*^2^ + 8*J*_2_*|S|*^2^ + 4*J*_2’_ *|S|*^2^ – *A|S|*^2^

*E_AFM-_*_2_ *= E*_0_ + 8*J*_1_*|S|*^2^ + 8*J*_2_*|S|*^2^ + 4*J*_2’_ *|S|*^2^ – *A|S|*^2^

*E_AFM-_*_3_ *= E*_0_ + 0*J*_1_*|S|*^2^ – 24*J*_2_*|S|*^2^ + 4*J*_2’_ *|S|*^2^ – *A|S|*^2^

*E_FM_ = E*_0_ – 16*J*_1_*|S|*^2^ – 8*J*_2_*|S|*^2^ – 8*J*_3_*|S|*^2^ – 4*J*_2’_ *|S|*^2^ – 4*J*_3’_ *|S|*^2^ – *A|S|*^2^

*E_AFM-_*_0_ *= E*_0_ – 16*J*_1_*|S|*^2^ – 8*J*_2_*|S|*^2^ + 8*J*_3_*|S|*^2^ – 4*J*_2’_ *|S|*^2^ + 4*J*_3’_ *|S|*^2^ – *A|S|*^2^

*E_AFM-_*_1_ *= E*_0_ – 0*J*_1_*|S|*^2^ – 8*J*_2_*|S|*^2^ + 8*J*_3_*|S|*^2^ + 4*J*_2’_ *|S|*^2^ – 4*J*_3’_ *|S|*^2^ – *A|S|*^2^

*E_AFM-_*_2_ *= E*_0_ – 0*J*_1_*|S|*^2^ – 8*J*_2_*|S|*^2^ + 8*J*_3_*|S|*^2^ + 4*J*_2’_ *|S|*^2^ + 4*J*_3’_ *|S|*^2^ – *A|S|*^2^

*E_AFM-_*_3_ *= E*_0_ + 16*J*_1_*|S|*^2^ – 8*J*_2_*|S|*^2^ + 8*J*_3_*|S|*^2^ – 4*J*_2’_ *|S|*^2^ + 4*J*_3’_ *|S|*^2^ – *A|S|*^2^

*E_AFM-_*_4_ *= E*_0_ – 8*J*_1_*|S|*^2^ + 0*J*_2_*|S|*^2^ + 0*J*_3_*|S|*^2^ – 4*J*_2’_ *|S|*^2^ + 4*J*_3’_ *|S|*^2^ – *A|S|*^2^

where E_0_ is the energy of the nonmagnetic state, *J*_1_ is 1^st^ neighbor exchange coupling parameters between surface transition metal atom M1 and middle layer transition metal atom M2, *J*_2_, *J*_2’_ *J*_3_, *J*_3’_ are 1^st^ and 2^ed^ neighbor exchange coupling parameters in surface layer and middle layer, respectively. A is anisotropy energy parameter, which is obtained by using the magnetic anisotropy energy as:

A = $\frac{E_{hard}\left( axis \right)-E_{easy}(axis)}{{|S|}^{2}}$

Table S5. Calculate results of magnetic moments (μB) and Bader charge (in electron) on M1 and M2 atom for *h*-M_3_B_4_ and *ort*-M_3_B_4_ (M = Cr, Mn, Fe).

|  | *M* of M1(μB) | *M* of M2(μB) | ΔQ of M1(\|e\|) | ΔQ of M2(\|e\|) |
| --- | --- | --- | --- | --- |
| *h*-Cr_3_B_4_ | 3.55 | 2.49 | -0.59 | -0.97 |
| *h*-Mn_3_B_4_ | 3.97 | 3.25 | -0.57 | -0.92 |
| *h*-Fe_3_B_4_ | 2.26 | 1.04 | -0.41 | -0.51 |
| *ort*-Cr_3_B_4_ | 3.22 | 2.52 | -0.78 | -0.98 |
| *ort*-Mn_3_B_4_ | 3.64 | 2.82 | -0.77 | -0.90 |

Table S6. The total spin moment and orbital components projected on the s, p, d orbitals of 2D *ort*-Cr_3_B_4._ The unit of spin moments is µB.

| Atoms | *s* | *p* | *d* | total |
| --- | --- | --- | --- | --- |
| Cr1 | 0.026 | 0.038 | 3.153 | 3.217 |
| Cr2 | 0.026 | 0.038 | 3.153 | 3.216 |
| Cr3 | 0.012 | 0.016 | 2.486 | 2.515 |
| B1 | -0.018 | -0.070 | 0.000 | -0.088 |
| B2 | -0.018 | -0.070 | 0.000 | -0.088 |
| B3 | -0.032 | -0.112 | 0.000 | -0.144 |
| B4 | -0.032 | -0.112 | 0.000 | -0.144 |

Table S7. The magnetic anisotropy energy (MAE) and easy axis of ort-Cr_3_B_4_.

| *ort*-Cr_3_B_4_ | MAE (μeV) | easy axis |
| --- | --- | --- |
| 5×5×1 | 19 | *b* |
| 6×6×1 | 28.23 | *b* |
| 7×7×1 | 25.67 | *b* |

Table S8. Magnetic anisotropy energy (MAE) is defined as the energy difference between the system with spin direction along the magnetic hard axis and the system with spin parallel to the magnetic easy axis. K_1_ and K_2_ are anisotropy constants.

|  | K_1(a-c)_ | K_2(a-c)_ | K_1(b-c)_ | K_2(b-c)_ | MAE | Δ*E_SOC_* | easy axis |
| --- | --- | --- | --- | --- | --- | --- | --- |
| *h*-Cr_3_B_4_ | 15.471 | -0.249 | 28.35 | 0.701 | 29.05 | 66 | *c* |
| *h*-Mn_3_B_4_ | 165.548 | 1.476 | 172.68 | -0.652 | 172.02 | 393 | *c* |
| h-Fe_3_B_4_ | 138.997 | 2.494 | 248.89 | 5.775 | 254.17 | 496 | c |
| *ort*-Cr_3_B_4_ | 8.577 | -1.278 | -19.69 | -1.1578 | 28.23 | -39 | *b* |
| *ort*-Mn_3_B_4_ | 174.483 | 0.129 | 197.13 | 0.137 | 197.26 | 379 | *c* |
